# Amputations of Thigh and Leg

**Published:** 1889-03

**Authors:** Mordecai Price

**Affiliations:** Philadelphia


### Selections.

### *AMPUTATIONS OF THIGH AND LEG.**

By MORDECAI PRICE, M. D., of Philadelphia.

The subject I wish to bring before the Society to-night is one of deep interest—to me at least, and to all others who are alike unfortunate in having lost a limb. In performing amputation on the leg, the chief object of the surgeon of the day seems to be to remove the limb and save life—the future comfort and usefulness of the patient being minor considerations. The comfort and usefulness of patients who are subjected to amputation of the leg have received my personal attention through the entire period of my professional life. This has been my latest thought at night; my first consideration in the morning; and I have been painfully reminded many times during the day of the importance of changes in the present surgical practice. I see no reason why in this department we should narrow our surgery to a strict following of the *dictum* of a school of surgeons a century old; why we should not, in step with other departments of surgery and medicine, adopt those new truths which our advanced art and science, and wider experience approve.

I ask your critical consideration of the few changes I propose to suggest. As a student, I often marveled at the numerous amputations done near the ankle and through the knee, for the reason then given: to save all the limb possible, apparently without due consideration of the discomfort and suffering to follow, and the usefulness of the limb. I have waited for years hoping that some of our eminent surgeons, members of this Society, would bring the subject before us, when I expected to be able to say something upon the subject. Like many other departments of medicine and surgery, however, this seems to

* Read before the Philadelphia County Medical Society, January 23, 1889.

have been looked upon as one of the subjects forever settled. For, as far back as the works of surgery of the eighteenth century, I find the same old plates, the same old positions for the removal of the limb—where it is a matter of selection with the surgeon—as I find in use to-day. Sometimes the accident comes to the patient's rescue and removes sufficient of the limb to compel the surgeon to give the patient a good stump. In an amputation to-day in the foremost hospital of the world—the Pennsylvania—if the location of the injury left a choice as to where the limb should be removed, it would be done through the ankle or at the lower third of the leg. I suppose you ask "Why not?" I would answer that question by asking the question: Why do we amputate at all? The answer would be: first, to save life; and second, to make a useful limb. Now, we can save life as easily by one method as by the other. Why not, then, operate solely for the best interest of the patient? In an amputation of the leg, all that is left below the middle of the middle third of the leg is useless and in the way, and gives that much more room for ulceration and friction sores. Let me tell you, gentlemen, these are weighty considerations in an amputation, for they compel the wearer of an artificial limb either to endure great suffering or to leave the artificial limb off, as I can abundantly testify from personal experience.

Nearly three-quarters of a century ago, Gibson used the following language: "As much as possible of the thigh should in all cases be saved. But the rule does not always hold good in amputations of the leg. If, for example, the leg be amputated just above the ankle, the bone, from the deficiency of surrounding muscle, cannot be well covered, and is, therefore, not calculated to bear the pressure of an artificial leg. On this account, the patient is obliged to have an instrument of the kind adapted to the knee, and the leg, therefore, is carried out behind at right angles with the thigh, and by its weight greatly incommodes the patient; so much so, indeed, that I have known two or three to submit to a second operation, for no other reason than to get rid of the incumbrance." This Dr. Gibson gives as his professional experience. I personally know of a number of reamputations for no other reason than the suffering, discomfort, or absolute impossibility of wearing an artificial limb upon a long stump. After the application of an artificial limb, there is a constant diminution of the size of the stump. Its nutrition being continually interfered with, and the parts being of low vitality, consequently, when we have ulceration or friction sores of any kind, it is with great difficulty that they are induced to heal.

There is another element to be taken into consideration: As soon as the artificial limb is left off, and the patient assumes an upright position, the limb is greatly enlarged by a species of edema which takes place immediately, leaving the parts in no condition to heal. The limb has the feeling of being cold and almost lifeless; and if exposed to cold, it would be the first to freeze. It is almost impossible to keep the amputated limb warm.

When the artificial one is left off, amputations through the knee-joint give in many cases a very bad surface to bear the weight of the body, and a leg is rarely worn with comfort. Such an amputation absolutely prevents the application of a full-lengthed limb, as the knee-joint would have to be lowered some three inches for want of room, making at best a useless appliance. Amputate, therefore—if it is a matter of selection—through the lower third of the thigh. An amputation below the middle of the leg is objectionable on account of the length of the stump, which presents occasion for ulceration and is difficult to dress properly so that the limb may be worn with comfort. Every inch of stump over five or six inches below the knee involves that many hundreds of hours of suffering and distress to the patient. The additional chance of life does not add one feather's weight in favor of the long amputation. Amputation at the lower third does not give sufficient room for a strong ankle-joint, and, therefore, adds greatly to the wear and tear of the limb, thus adding largely to the expense. Amputations through the ankle may give the patient something to walk on, but this is oftentimes accompanied with great pain. It often gives him a poor excuse for a limb, and completely prevents any mechanical appliance from aiding him in the least, and forever prevents him from hiding his terrible deformity. If ever there was an appliance to which the term "slip-shod" could be appropriately applied, it is to those intended to imitate nature in these cases. The usefulness of an artificial limb is in proportion to the simplicity and completeness of its mechanical construction. The nearer it resembles the human limb in all its parts, the more perfectly it fills its office. There is one fact associated with these cases to which but few ot you, perhaps, have given a thought; that is the ever-present and painful consciousness of physical deformity which the patient has, and the fact that his maimed condition closes to him many avenues of honorable, useful, and lucrative employment. This applies especially to the case of civilians; to the soldier it is different; to him the loss of part of a limb is unchallenged testimony of gallant and heroic sacrifice.—*Maryland Medical Journal.*

